# Stable green phosphorescence organic light-emitting diodes with low efficiency roll-off using a novel bipolar thermally activated delayed fluorescence material as host†
†Electronic supplementary information (ESI) available: Physical properties of DPDDC, TGA and DSC, CV curves, absorption and emission spectra, PL decay curve, as well as device performances. See DOI: 10.1039/c6sc03008d



**DOI:** 10.1039/c6sc03008d

**Published:** 2016-10-04

**Authors:** Kunping Guo, Hedan Wang, Zixing Wang, Changfeng Si, Cuiyun Peng, Guo Chen, Jianhua Zhang, Gaofeng Wang, Bin Wei

**Affiliations:** a School of Mechatronic Engineering and Automation , Shanghai University , 149 Yanchang Road , Shanghai , 200072 , P. R. China . Email: bwei@shu.edu.cn; b Key Laboratory of Advanced Display and System Applications , Ministry of Education , Shanghai University , 149 Yanchang Road , Shanghai , 200072 , P. R. China . Email: zxwang78@shu.edu.cn; c Department of Chemistry , Shanghai University , 99 Shangda Road , Shanghai , 200444 , P. R. China; d Ningbo Intime Technology Co. Ltd , No. 23, Ruhu West Road, Simen Town , Yuyao City , Zhejiang Province 345403 , P. R. China

## Abstract

A novel bipolar hosting material, 11-(3-(4,6-diphenyl-1,3,5-triazin-2-yl)phenyl)-12,12-dimethyl-11,12-dihydroindeno[2,1-*a*]carbazole (DPDDC), was designed, synthesized, and characterized for green phosphorescent organic light-emitting diodes (PhOLEDs). The DPDDC exhibits excellent hole and electron transport properties, superior thermal stability, a high glass-transition temperature and a small singlet-triplet energy gap for efficient reverse intersystem crossing from triplet to singlet, reducing the triplet density of the host for PhOLEDs. The electrophosphorescence properties of the devices using DPDDC as the host and three green phosphorescent iridium(iii) complexes, bis(2-(4-tolyl)pyridinato-*N*,*C*2′)iridium(iii) acetylacetonate, bis(2-phenylpyridine)iridium(iii) acetylacetonate, and bis(4-methyl-2,5-diphenylpyridine)iridium(iii) acetylacetonate [(mdppy)_2_Iracac] as the emitter were investigated. The green PhOLED with 5 wt% (mdppy)_2_Iracac presents an excellent performance, including a high power efficiency of 92.3 lm W^–1^, high external quantum efficiency of 23.6%, current efficiency roll-off as low as 5.5% at 5000 cd m^–2^ and a twentyfold lifetime improvement (time to 90% of the 5000 cd m^–2^ initial luminance) over the reference electrophosphorescent device.

## Abstract 2

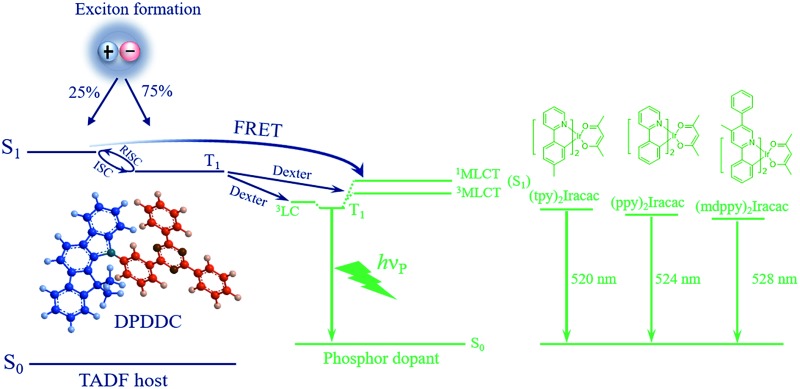
A bipolar host with TADF properties is realized, endowing green PhOLEDs with a twentyfold lifetime improvement.

## Introduction

Organic light-emitting diodes (OLEDs) have attracted intense interest owing to their potential applications in the fields of flexible flat-panel displays and solid-state lighting.^1–3^ Phosphorescent OLEDs (PhOLEDs) have been considered the ultimate technology, as they can give unity internal quantum efficiencies through harvesting both singlet and triplet excitons.^4–6^ Recently, green emission has almost approached the theoretical limitation of external quantum efficiency (EQE) by utilizing various dopants, such as *fac*-tris(2-phenylpyridinato)iridium(iii), as the phosphorescent emitter, the commercialization of which has been successfully started.^7–9^ However, typical PhOLEDs encounter significant roll-off (*i.e.*, current efficiency or EQE drastically drops as the applied voltage or brightness increases) problems, leading to an undesired low efficiency at high luminance, which is unfavorable for their commercial realization for lighting.^10,11^

Engineering designs for devices have been employed to enhance the high-brightness performance of PhOLEDs by reducing molecular aggregation, broadening the recombination zone, decreasing the exciton lifetime, and triplet management.^12^ Hao *et al.* had reported the accelerated triplet exciton diffusion used in stack emitting layer devices to be an effective strategy to reduce efficiency roll-off.^13^ Lee *et al.* reported green and red PhOLEDs with high efficiencies, low operating voltages, and low efficiency roll-offs with an exciplex forming co-host.^14^ Holmes *et al.* developed a graded dopant concentration profile in a broadened emissive layer, which provides a feasible approach to suppress triplet–triplet annihilation (TTA) and triplet–polaron annihilation (TPA).^15^ Nevertheless, the critical current density, representing the current density at which the EQE drops to half of its maximum value, is still low. Currently, intense research interest is still focused on highly efficient host materials.^16,17^ Commonly used hosts for phosphorescent emitters are conventional unipolar electron- or hole-transport materials, such as 1,3-bis(carbazolyl)benzene (mCP), *N*,*N*′-dicarbazolyl-4,4′-biphenyl (CBP) or *p*-bis(triphenylsilyl)benzene (UGH2), which have been used to achieve high EQEs above 20%.^18–20^ However, due to the large energy gap between the singlet (S_1_) and triplet (T_1_) energies of those compounds, a high T_1_ is always accompanied with an even higher S_1_, leading to a mismatch in the frontier energy levels with the adjacent function layers, and consequently, a high triplet exciton density.^21,22^ This influence is detrimental to the efficiency roll-off in PhOLEDs, in virtue of the TTA and TPA processes.^10,14,23^ A potential alternative is to use materials with thermally activated delayed fluorescence (TADF),^24–27^ as proposed by Adachi *et al.* ^28^ The triplet excitons are upconverted to the singlet state of the TADF molecules and then resonantly transferred to the singlet of the emitter molecules for light emission *via* the Förster resonant energy transfer (FRET) process. Very recently, Lee *et al.* used the exciplex with TADF properties as the host for the fluorescent emitters to achieve high performance OLEDs.^29^ These interesting results inspired us to use TADF molecules as the host for green PhOLEDs.

For practical applications, the light source requires a brightness of 1000–5000 cd m^–2^, a high power efficiency close to standard fluorescent lamps (40–70 lm W^–1^) and 10 000 hours of lifetime simultaneously.^30,31^ Unfortunately, typical OLEDs encounter the deleterious impact of water vapor and oxygen, leading to unacceptably short operation lifetimes, and this has prevented their application in displays for more than decade.^32^ Thus, an OLED device with a longer lifetime at high-brightness levels is also highly expected.

In this paper, we report an excellent bipolar host material of green PhOLEDs with TADF properties, 11-(3-(4,6-diphenyl-1,3,5-triazin-2-yl)phenyl)-12,12-dimethyl-11,12-dihydroindeno[2,1-*a*]carbazole (DPDDC), in which the hole-transporting indenocarbazole and electron-withdrawing 2,4-diphenyl-1,3,5-triazine units were connected with the unconjugated benzene ring. DPDDC exhibited a small singlet-triplet energy gap (Δ*E*_ST_) of 0.18 eV, affording efficient reverse intersystem crossing (ISC), and TADF features. Three phosphorescent guests, bis(2-(4-tolyl)pyridinato-*N*,*C*2′) iridium(iii) acetylacetonate [(tpy)_2_Iracac], bis(2-phenylpyridine)iridium(iii) acetylacetonate [(ppy)_2_Iracac], and bis(4-methyl-2,5-diphenylpyridine)iridium(iii) acetylacetonate [(mdppy)_2_Iracac] were doped in the DPDDC host and the device performances were investigated. High efficiency green PhOLEDs with maximum EQEs of 18.1%, 22.6% and 23.6% were achieved, and the efficient reverse ISC of DPDDC endowed the devices with low efficiency roll-off behavior by reducing the triplet density on the host and thus diminishing the TTA and TPA. The (tpy)_2_Iracac, (ppy)_2_Iracac, and (mdppy)_2_Iracac-based devices showed extremely low current efficiency roll-offs of 2.3%, 5.2%, and 5.5% at 5000 cd m^–2^, respectively. The best PhOLED also presented a high power efficiency of 92.3 lm W^–1^ (84.3 cd A^–1^) and a sixfold operation lifetime improvement under air conditions compared to CBP-based devices under a nitrogen atmosphere.

## Results and discussion

### Synthesis and characterization

**Scheme 1:**
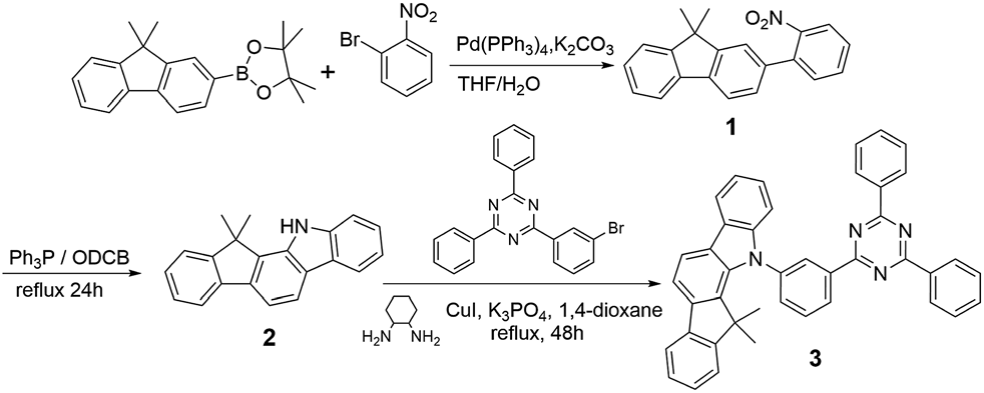
The synthetic process for DPDDC.

The synthetic routes to the host material DPDDC are illustrated in Scheme 1. The synthesis of intermediate 12,12-dimethyl-11,12-dihydroindeno[2,1-*a*]carbazole was followed from a literature procedure.^33^ Compound **2** was treated with 2-(3-bromophenyl)-4,6-diphenyl-1,3,5-triazine under Ullmann reaction conditions to produce compound **3** in a 45% yield. The details for the preparation of these compounds are given in the Experimental section.

**Fig. 1:**
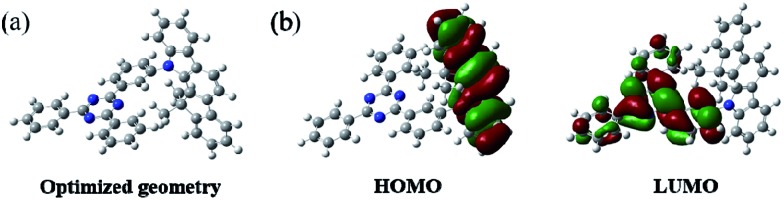
The optimized geometry and the molecular orbital surface of the HOMO and LUMO for DPDDC obtained at the B3LYP/6-31G level.

In order to understand the structural properties of our designed host material, the three-dimensional geometry and the frontier molecular orbital energy levels of DPDDC were calculated using density functional theory (DFT) at the B3LYP/6-31G level. Fig. 1b depicts the molecular orbital dispersion of DPDDC in the highest occupied molecular orbital (HOMO) and the lowest unoccupied molecular orbital (LUMO) states. Similar to the reported TADF emitters and bipolar phosphorescent hosts, the HOMO of DPDDC was mainly dispersed over the electron-donating indenocarbazole moiety, while its LUMO was localized on the electron-withdrawing 2,4-diphenyl-1,3,5-triazine moiety.^34,35^ The separated HOMO and LUMO contribute holes and electrons transferring balance. No obvious overlap between the HOMO and LUMO can be observed due to the effective separation of the electron densities of the HOMO and LUMO by the unconjugated fluorene moiety. This indicates a small energy difference between its singlet and triplet states.^28,36^ The thermal stability of DPDDC was investigated by using thermal gravimetric analysis (TGA) and differential scanning calorimetry (DSC) under a nitrogen atmosphere (Fig. S1, in the ESI†). DPDDC exhibited a good thermal stability with a high decomposition temperature (*T*_d_) of 441 °C (corresponding to a 5% weight loss), and a high glass transition temperature (*T*_g_) of 140 °C (Table S1, in the ESI†), which are higher than the widely used CBP host material and the thioxanthone-based TADF emitter.^35,37^ Such excellent thermal stability is beneficial to form homogeneous and amorphous films with high morphological stability.

### Electrochemical and photophysical properties

The electrochemical properties of DPDDC were determined using cyclic voltammetry (CV) and differential pulse voltammetry (DPV), and the redox potentials were reported relative to a ferrocenium/ferrocene (Fc^+^/Fc) redox couple as an internal standard.^38^ The oxidation potential of DPDDC was at 1.02 V which was contributed to the indeno[2,1-*a*]carbazole unit, and the reduction potential was at –2.73 V which was contributed to the 2,4,6-diphenyl-1,3,5-triazine unit. From the oxidation onset potential, the HOMO energy level of the DPDDC was estimated to be –5.82 eV, while the LUMO energy level was calculated to be –2.57 eV from the absorption edge of the optical absorption spectra. As expected, the experimental HOMO and LUMO values are in excellent agreement with the calculated values (Table S1, ESI†).

**Fig. 2:**
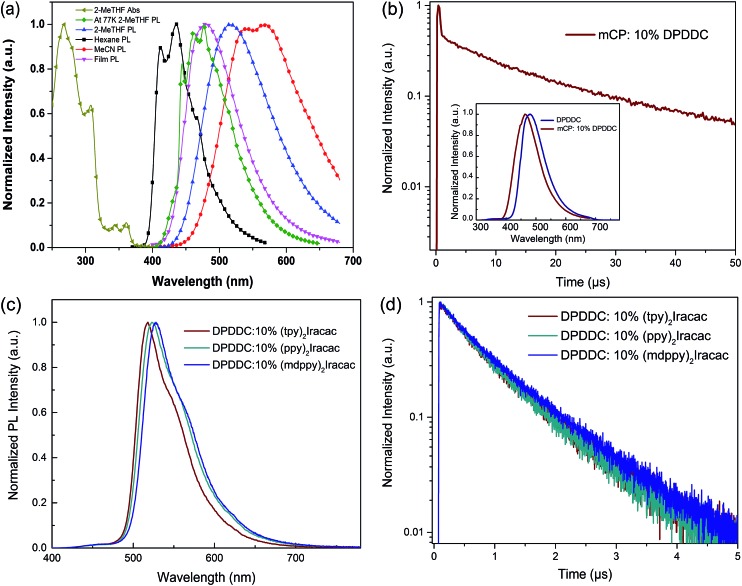
(a) UV-vis absorption (Abs) and PL spectra of DPDDC in diluted solution (hexane, 2-MeTHF, and MeCN) and in thin solid film at room temperature and low temperature; (b) the transient decay curve of mCP: 10 wt% DPDDC film at room temperature. Inset: PL spectra of neat DPDDC and mCP:DPDDC films. (c) PL emission spectra (d) and PL transient decay curves of Ir-doped DPDDC films.

The ultraviolet-visible (UV-vis) absorption and photoluminescence (PL) emission spectra of DPDDC are shown in Fig. 2a. The compound DPDDC exhibited π–π* transitions below 315 nm, and n–π* transitions between 315 and 370 nm in the UV-vis absorption spectra. The maximum PL emission wavelengths of DPDDC were observed at 415, 515, and 540 nm in hexane, 2-methyltetrahydrofuran (2-MeTHF), and acetonitrile (MeCN) solution (10^–5^ M), respectively, at room temperature. Comparing with its UV-vis absorption spectra, the big Stokes shifts were observed in different solutions, which means that compound DPDDC has more geometrical change and intramolecular charge transfer in the excited state. It showed the maximum PL emission peak at 475 nm in the thin solid film. The phosphorescence spectrum of DPDDC was measured in oxygen-free 2-methyl THF solution at low temperature (77 K, in liquid nitrogen) and the triplet energy was estimated to be 2.79 eV from the highest energy vibronic band. Thus, the Δ*E*_ST_ can be estimated to be 0.18 eV, which is relatively small given that the Δ*E*_ST_ of most hosts reported are in the range of 0.5–1.0 eV.^39^ The S_1_ and T_1_ energies are summarized in Table S2 (in the ESI†). The Δ*E*_ST_ of the DPDDC is very low compared to that of CBP, which contributes to the efficient RISC from T_1_ to S_1_.

To demonstrate the TADF properties of DPDDC, the transient PL decay of the doped film of DPDDC in mCP was investigated. A doped film instead of a neat film is used to suppress concentration quenching, and the doping concentration is 10 wt%. As shown in Fig. 2b, the transient decay curve can be resolved into two components: the prompt component and the delayed component (see Fig. S2, ESI†). For the doped film, the transient decay times of the prompt component and the delayed component were estimated to be 46.4 ns and 12.3 μs from a second-order exponential decay fitting. Thus, the prompt fluorescence can be assigned to the fluorescence of DPDDC and the delayed fluorescence can be attributed to the TADF occurring *via* reverse ISC, which coincides with the small Δ*E*_ST_ value of DPDDC. Interestingly, the spectra of the doped and undoped films were slightly different according to the inset of Fig. 2b, which was ascribed to the intermolecular interactions in the neat film.

Considering the high triplet energy and suitable frontier energy levels of DPDDC, we were interested in the application of DPDDC as the matrix material of PhOLEDs by using a series of green emitters, (tpy)_2_Iracac, (ppy)_2_Iracac and (mdppy)_2_Iracac as dopants. The PL emission of DPDDC overlaps well with the UV-vis absorption of the metal to ligand charge transfer (MLCT) for the three Ir-complexes, suggesting efficient Förster energy transfer from DPDDC to the emitters (Fig. S3, in the ESI†). Fig. 2c shows the PL spectra of 10 wt% Ir-complex doped films of the DPDDC. The PL spectra from the doped films are dominated by a band centered near 520 nm, which gives a lifetime of transient decay in the microsecond regime (Fig. 2d). The PL quantum yields (PLQYs) of (tpy)_2_Iracac, (ppy)_2_Iracac and (mdppy)_2_Iracac from the DPDDC doped films were 79.3%, 85.6% and 89.5%, implying their promising applications in electroluminophores.

### Electroluminescence of the OLEDs

**Fig. 3:**
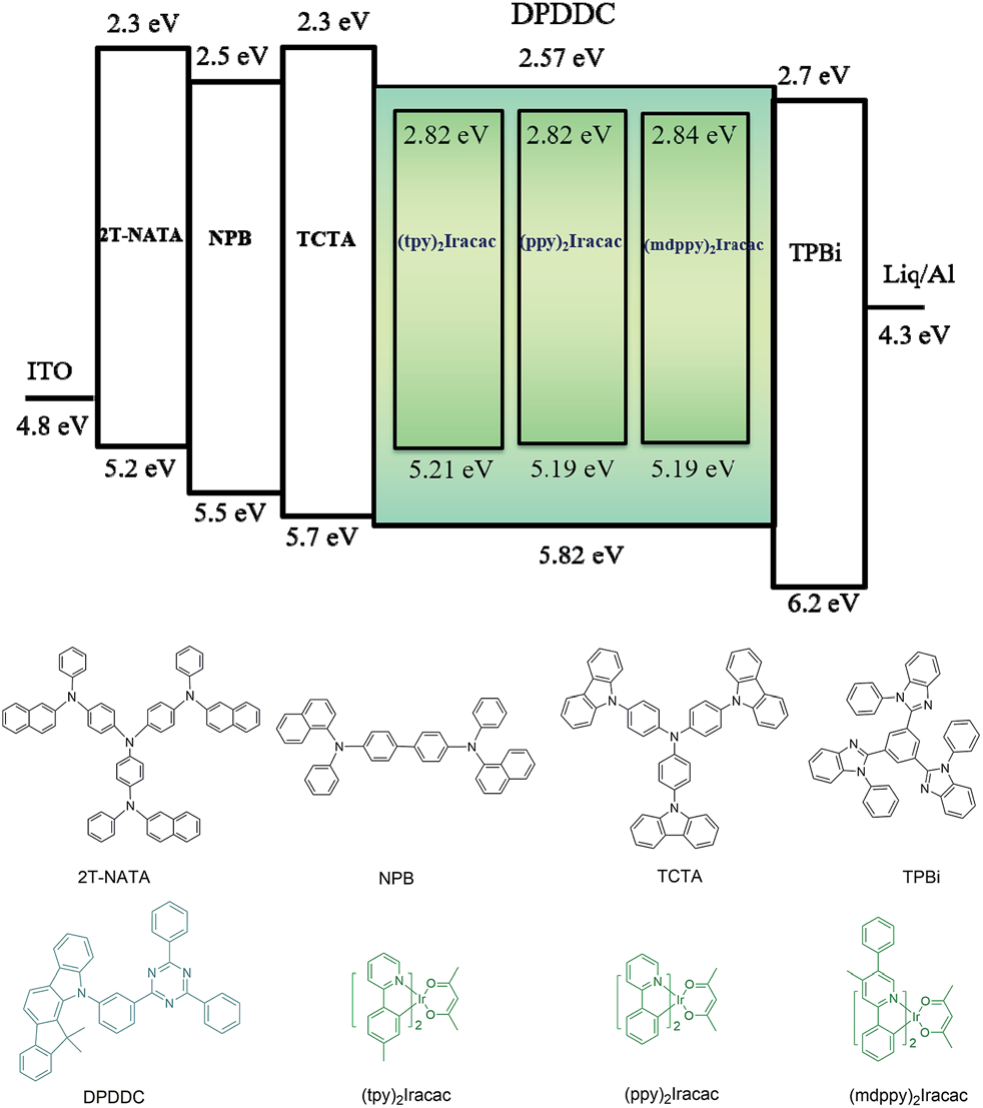
Device structure and chemical structures of the compounds used in the OLEDs.

In order to evaluate the capability of DPDDC as a host material in green PhOLEDs and to reveal the influence of the TADF properties on the device performance, a group of green-light emitting devices was fabricated. Ir-complex (tpy)_2_Iracac, (ppy)_2_Iracac and (mdppy)_2_Iracac were separately doped in DPDDC, and the molecular structures and energy levels used are illustrated in Fig. 3. The OLED structure was: indium tin oxide (ITO)/2T-NATA (25 nm)/NPB (5 nm)/TCTA (10 nm)/10 wt% (tpy)_2_Iracac:DPDDC (device A-1) or 10 wt% (ppy)_2_Iracac:DPDDC (device B-1) or 10 wt% (mdppy)_2_Iracac:DPDDC (device C-1) (20 nm)/TPBi (30 nm)/Liq (1 nm)/Al (100 nm), where 4,4′,4′′-tris-*N*-naphthyl-*N*-phenylamino-triphenylamine (2T-NATA) was employed as the hole injection layer (HIL), *N*,*N*′-bis-(naphthyl)-*N*,*N*′-diphenyl-1,1′-biphenyl-4,4′-diamine (NPB) and 4,4′,4′′-tris(*N*-carbazolyl)-triphenyl-amine (TCTA) were used as the hole transport layers (HTLs), and 1,3,5-tris(*N*-phenylbenzimidazol-2-yl) benzene (TPBi) was applied to the electron transport layer (ETL). The introduction of the TCTA and TPBi layers did not only transport carriers effectively, but also plays a significant role in blocking carriers, by which we confine excitons in the EML for avoiding the energy outflow.^40^ Besides, as a commonly used host for green phosphorescent emitters, CBP was chosen for comparison, referred to as devices A-2, B-2 and C-2 for the (tpy)_2_Iracac, (ppy)_2_Iracac and (mdppy)_2_Iracac emitters, respectively.

**Fig. 4:**
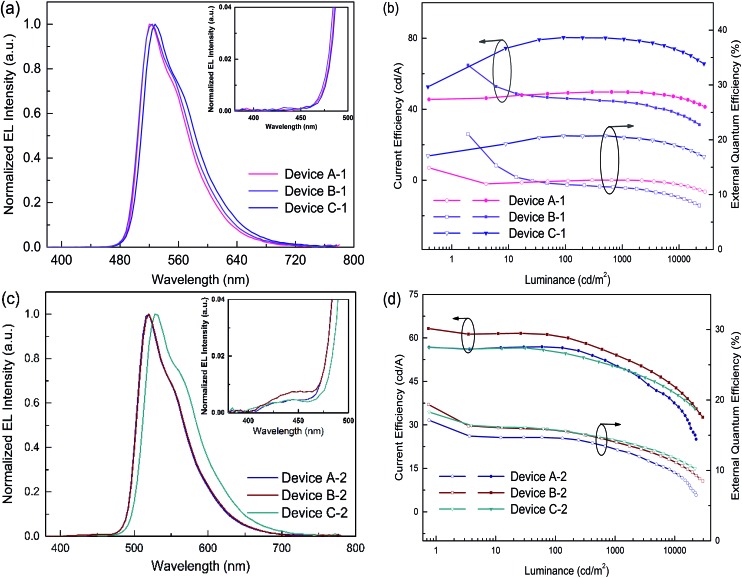
(a) EL spectra under a luminance of 1000 cd m^–2^ and (b) Ce–*L*–EQE characteristics of devices A-1, B-1 and C-1. (c) EL spectra under the luminance of 1000 cd m^–2^ and (d) Ce–*L*–EQE characteristics of devices A-2, B-2 and C-2. Inset of (a and c) shows magnified band edge between 380 and 500 nm.

All of the PhOLEDs emitted green light with the EL emission peaks at 520, 528 and 532 nm for devices A-1, B-1 and C-1, respectively. As shown in Fig. 4a, the EL spectra of all the PhOLEDs exhibited dominant emission bands corresponding to the iridium(iii) complex without any other residual emission band originating from the host and/or adjacent layers, suggesting that the excitons generated in the emissive layer were mainly activated by the phosphorescence emission of the iridium complexes. The Commission Internationale d'Eclairage (CIE) color coordinates of all the PhOLEDs were in the green region of the CIE chromaticity diagram (Fig. S4, in the ESI†). Moreover, it was found that the EL spectra of the DPDDC-based devices revealed almost identical behavior to CBP-based devices, as shown in Fig. 4. However, it is worth noting that a slight blue emission with a peak wavelength of 450 nm was observed for the CBP-based devices, which truly originated from the exciplex emission of CBP/TPBi.^41^

The current density–voltage (*J*–*V*) and luminance–voltage (*L*–*V*) characteristics of the devices are shown in Fig. S5 (in the ESI†), and the key device performance parameters are summarized in Table 1. Devices A-1, B-1 and C-1 have low turn-on voltages of 2.6, 2.7 and 2.6 V, respectively, at a luminance of 1 cd m^–2^. While the CBP-based green devices indicated quite high turn-on voltages around 3.0 V (see Table 1). In the whole operating voltage range, DPDDC-based devices always delivered higher current density than CBP-based devices at a given voltage. The higher current density in DPDDC-based devices should result from the facilitated electron injection and transportation. According to the energy level diagram in Fig. S6 of ESI,† the electron injection barrier at the DPDDC/TPBi interface (0.13 eV) is much lower than that at the CBP/TPBi interface (0.43 eV), which is definitely helpful to enhance electron injection into the emitting layer and result in the lower driving voltages in DPDDC-based devices.

**Table 1:** The performance summary of the PhOLEDs based on DPDDC and common CBP hosts

	Device	Host	*V*_on_ ^*a*^ (V)	*η*_maxCe_ (cd A^–1^)	*η*_Ce_ ^*b*^ (cd A^–1^)	*η*_Ce_ ^*c*^ (cd A^–1^)	*η*_Ce_ ^*d*^ (cd A^–1^)	*η*_maxEQE_ (%)	*η*_EQE_ ^*b*^ (%)	*η*_EQE_ ^*c*^ (%)	*η*_EQE_ ^*d*^ (%)	*η*_maxPe_ (lm W^–1^)	CIE^*c*^ (*x*, *y*)
10% Ir-complex based device	A-1	DPDDC	2.6	49.7	49.1	49.7	48.3	14.9	12.5	12.6	12.3	55.0	(033, 0.62)
	B-1	DPDDC	2.7	64.8	46.1	44.2	40.3	21.0	11.8	11.3	10.3	75.4	(036, 0.61)
	C-1	DPDDC	2.6	80.4	80.4	79.5	76.2	20.7	20.7	20.5	19.6	83.3	(0.38, 0.60)
	A-2	CBP	3.1	56.9	56.7	50.7	41.8	17.1	14.6	13.0	10.9	57.6	(0.32, 0.63)
	B-2	CBP	3.1	63.1	60.8	54.3	47.3	19.4	15.7	14.1	12.3	63.9	(0.32, 0.63)
	C-2	CBP	2.9	56.6	55.3	50.2	44.5	18.3	15.7	14.3	12.7	61.3	(037, 0.60)
5% Ir-complex based device	A-3	DPDDC	2.7	70.8	66.0	70.4	69.2	18.1	16.9	18.0	17.7	67.4	(032, 0.63)
	B-3	DPDDC	2.7	73.4	73.2	72.8	69.6	22.6	18.6	18.5	17.7	77.0	(035, 0.61)
	C-3	DPDDC	2.6	84.3	84.3	83.4	79.7	23.6	21.8	21.5	20.6	92.3	(0.37, 0.60)

^*a*^Measured at a luminance of 1 cd m^–2^.

^*b*^Measured at a luminance of 100 cd m^–2^.

^*c*^Measured at a luminance of 1000 cd m^–2^.

^*d*^Measured at a luminance of 5000 cd m^–2^.

Accordingly, the DPDDC-based devices achieved higher efficiencies. The current efficiency–luminance–external quantum efficiency (Ce–*L*–EQE) characteristics are shown in Fig. 4b. The power efficiency–luminance (Pe–*L*) curves can be found in Fig. S7 (ESI†). Device C-1 reached the highest efficiency of 80.4 and 79.5 cd A^–1^, and a beyond theoretical limit EQE of 20.7 and 20.5%, at a luminance of 100 and 1000 cd m^–2^, respectively. Importantly, a high efficiency of 76.2 cd A^–1^ (19.6% EQE) even at 5000 cd m^–2^ was also obtained, which signifies a mild efficiency roll-off. While in the case of (tpy)_2_Iracac-based device A-1 and (ppy)_2_Iracac-based device B-1, though low roll-off at high luminance, the efficiencies were still low (Ce of 49.7 and 44.2 cd A^–1^ at 1000 cd m^–2^ for devices B-2 and C-2). The reason for the lower efficiencies of devices A-1 or B-1 than device C-1 may be attributed to a non-efficient host–guest energy transfer.

The EL processes of the DPDDC-based devices are illustrated in Fig. S8 (see ESI†): (i) the S_1_ and T_1_ excitons which are electrically generated mainly formed on the DPDDC host. (ii) The S_1_ excitons then have three possible decay pathways: radiative decay from the S_1_ state to the ground state (S_0_) with a radiative decay rate of *κ*Sr = 8.4 × 10^5^ s^–1^; ISC back to the T_1_ state of DPDDC with a rate constant of *κ*_ISC_ = 2.0 × 10^7^ s^–1^; and FRET from the S_1_ state of DPDDC to the ^1^MLCT state of the Ir-complex. The rate constant of FRET could be estimated: *κ*_ET_ = (1/*τ*_D_)(*R*60/*R* ^6^), where *τ*_D_ is the radiative decay time of the donor molecule, *R*_0_ is the Förster transfer radius, and *R* is the average distance between the host and dopant molecules. (iii) Meanwhile, the T_1_ excitons on DPDDC also have several possible decay pathways: it promptly converts to the S_1_ state through RISC with a rate constant of *κ*_RISC_ = 7.6 × 10^5^ s^–1^ due to the small Δ*E*_ST_ = 0.18 eV (see ESI note†); Dexter energy transfer from the T_1_ state of DPDDC to the ^3^MLCT and ^3^LC state of the Ir-complex. For Ir-complex doped DPDDC, *R*_0_ is around 2 nm, *R*_0_ is around 2 nm (ref. 42) and *R* is estimated to be around 2.2 nm for the dopant concentration of 5–10 wt% using a molecular modeling program. Thus, the rate constant of FRET is around 10^9^ s^–1^ which is nearly two orders of magnitude faster than those of the ISC and RISC processes. The fast FRET will convert the electrically generated S_1_ excitons of DPDDC to the ^1^MLCT state of the Ir-complex. (iv) The MLCT state and ^3^LC state undergo ISC to form the lowest excited state of the dopant and radiative decay a phosphorescence emission. This can decrease the S_1_ density of DPDDC, thus reducing the ISC process of DPDDC from S_1_ to T_1_. Furthermore, the RISC of the DPDDC host from T_1_ to S_1_ could be enhanced, leading to the decreasing T_1_ concentration on the DPDDC host, thus lowering the efficiencies roll-off of the corresponding devices.^25^ The results showed that the device based on the (mdppy)_2_Iracac:DPDDC as an EML exhibited an EQE exceeding 20% and very-low efficiency roll-off. This shows that the RISC is not conflicting with the excellent device performance.

**Fig. 5:**
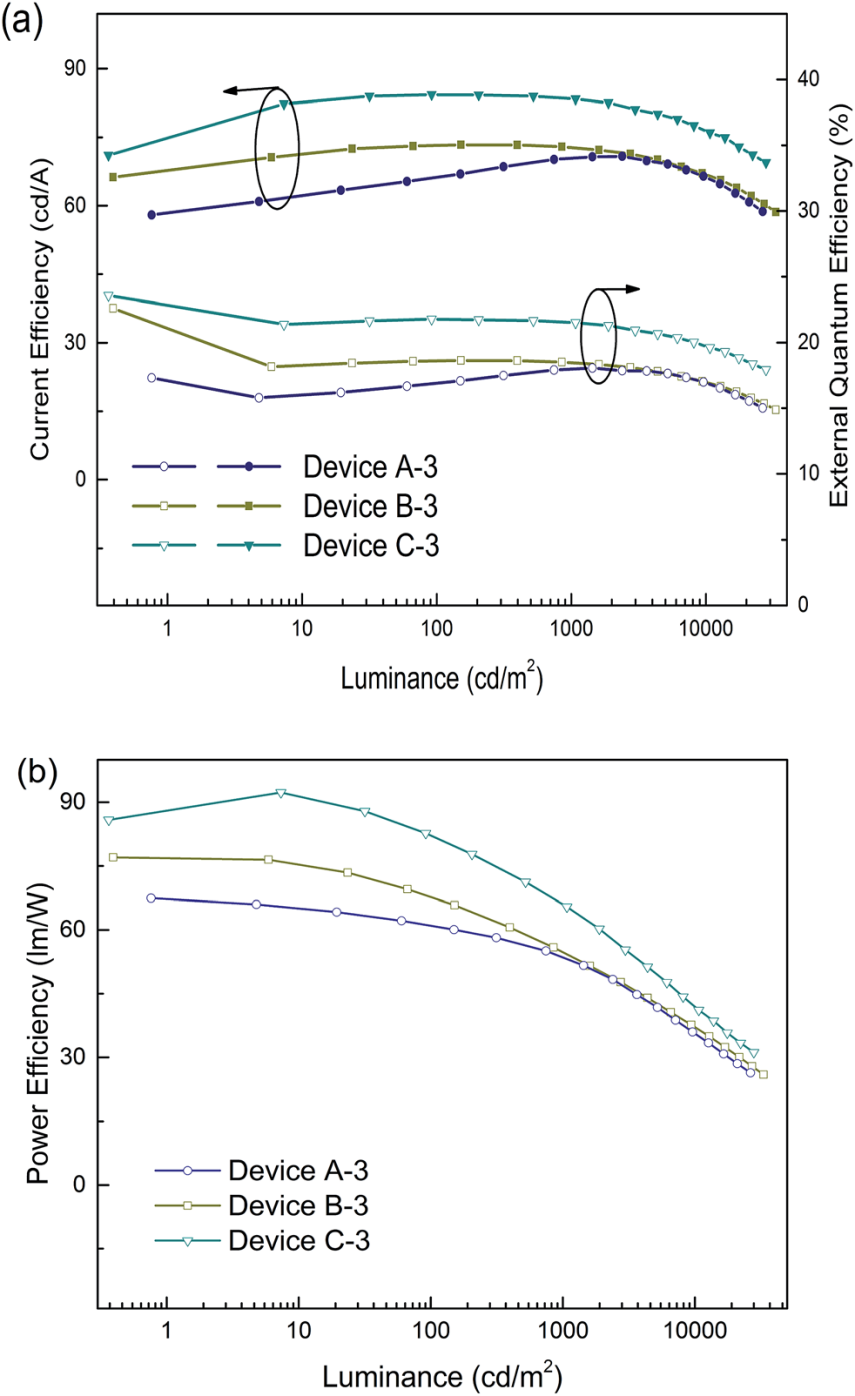
(a) Current efficiency and power efficiency plotted against luminance, and (b) the external quantum efficiency plotted current density for devices based on a 5 wt% Ir-complex doped host.

The emission efficiency of an OLED is dependent not only on the hosting matrix, but also on the dopant concentration. A high doping concentration of Ir-complex favors an efficient energy transfer, while it easily leads to exciton aggregation and serious self-quenching of the Ir-complex. In contrast, a low doping concentration of Ir-complex limits radioactive recombination on dopant sites, and results in inadequate utilization of excitation energy of the matrix.^19,43^ We further fabricated three devices with a 5 wt% doping concentration, referred to as devices A-3, B-3 and C-3 for the (tpy)_2_Iracac, (ppy)_2_Iracac and (mdppy)_2_Iracac emitters. A most remarkable observation from Fig. 5 is that ultra-high efficiencies were achieved for device C-3. The device afforded a maximum current efficiency of 84.3 cd A^–1^, a maximum power efficiency of 92.3 lm W^–1^, and a maximum external quantum efficiency of 23.6% without any light out-coupling enhancement. As expected, all devices showed extremely low efficiency roll-off. For device C-3, the Ce can be up to 83.4 cd A^–1^ with an efficiency roll-off of 1.1% at the luminance up to 1000 cd m^–2^ and 79.7 cd A^–1^ with an efficiency roll-off at 5.5% at the luminance up to 5000 cd m^–2^. Similar excellent performance can also be obtained for devices A-3 and B-3. The devices based on (tpy)_2_Iracac and (ppy)_2_Iracac indicated a very low efficiency roll-off of 2.3% and 5.2% at 5000 cd m^–2^ (Table 1), respectively. The Ir-complexes were surrounded by DPDDC under 5 wt% dopant concentration. For the ideal Dexter energy transfer, a close contact between host and dopant would significantly enhance the Dexter process. Complex (mdppy)_2_Iracac has two extra phenyl groups than (ppy)_2_Iracac, which could act as an antennae merging with DPDDC with closer van der Waals interactions and even better π–π interactions for energy transfer, resulting in the best efficiency among those three complexes. On the contrary, extra methyl groups of (tpy)_2_Iracac reduce the π–π interactions between (tpy)_2_Iracac and DPDDC, leading to the lowest efficiency of the device.

Compared with others, the efficiency of our devices are among the highest and the roll-off of our devices are significantly reduced, demonstrating that the DPDDC is a promising host for green PhOLEDs. We strongly believe that such outstanding performances can be attributed to the small Δ*E*_ST_ of DPDDC for efficient reverse ISC from triplet to singlet, and thus subdued TTA and TPA on host. More importantly, the DPDDC matrix provides desirable hole and electron transport, and proper charge balance at the emissive layer. Thus, charge carrier transport properties of bipolar DPDDC have been performed. In order to investigate the bipolar charge carrier transport characteristics of the host material, hole-only and electron-only devices were fabricated. The configuration of the hole-only device is ITO/NPB (60 nm)/host (30 nm)/NPB (40 nm)/Al (100 nm), and the electron-only device is ITO/TPBi (60 nm)/host (30 nm)/TPBi (40 nm)/Liq (1 nm)/Al (100 nm). The *J*–*V* characteristics of the hole-only and electron-only devices are shown in Fig. S9 (see ESI†). The appropriate hole and electron current densities in the only devices provide the evidence of bipolar characteristics in the DPDDC host material. On the other hand, CBP shows a slightly higher electron current density but a poor hole density, which clearly indicates the improper charge balance.

We have furthermore used neat DPDDC as an emitter to fabricate fluorescent OLEDs (device D). Fig. S10 of the ESI† shows the device performance of device D with undoped DPDDC EML. The highest current efficiency of 4.3 cd A^–1^ and the maximum power efficiency of 3.0 lm W^–1^ (3.3 mA cm^–2^) are obtained. The efficient sky-blue emission with a peak at *λ*_peak_ = 476 nm is observed at 1000 cd m^–2^, which is consistent with that of the PL emission. The CIE color coordinates of the sky-blue OLED are (0.22, 0.34) at 1000 cd m^–2^, corresponding to the sky-blue region in the CIE chromaticity diagram.

### High operational stability

**Fig. 6:**
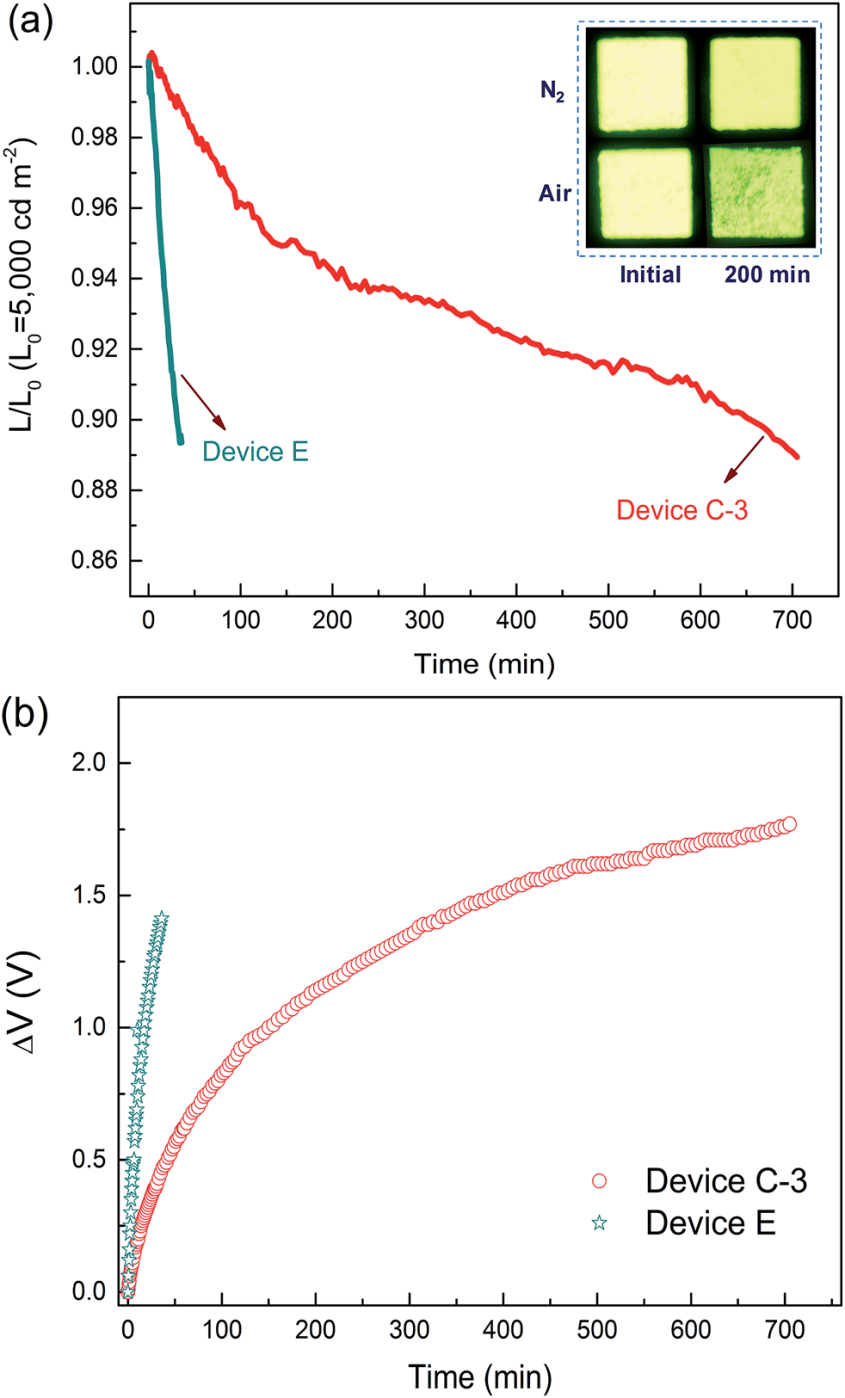
Time evolution of the normalized luminance, *L*, of devices C-3 and E in a dry-nitrogen (<1 ppm H_2_O and O_2_) atmosphere (a) and change in operating voltage Δ*V* (offset to zero) at the initial luminance of *L*_0_ = 5000 cd m^–2^ (b). The inset of (a) shows the photos of the test device C-3 after 200 min.

As mentioned above, standard OLEDs show a significant decrease in efficiency and lifetime under use in general lighting. Thus, the operation lifetime of device C-3 was measured to study the stability of the DPDDC host material. Additionally, the CBP-based reference device (device E) with 5 wt% (mdppy)_2_Iracac was also demonstrated. Fig. 6 shows the time evolution of the luminance, *L*, and the change in voltage from its initial value, Δ*V* = |*V*(*t* = 0) – *V*(*t*)|, for devices C-3 and E under conditions of air or nitrogen. The devices are operated at room temperature and at a constant current density for an initial luminance of 5000 cd m^–2^. As observed in Fig. 6a, the device C-3 shows a long lifetime of approximately 665 min up to 90% of initial luminance in a dry nitrogen atmosphere, which is more than 20 times longer than that of device E (*T*_90_ = 31 min) under a nitrogen atmosphere. To our knowledge, devices based on a TADF host at high-brightness levels rarely achieve an operation lifetime above 600 min.^44,45^ Furthermore, we also measured the lifetime of device C-3 in a humid air atmosphere with 20% relative humidity (Fig. S11, in the ESI†). Surprisingly, device C-3 exhibited a higher air stability with *T*_90_ = 201 min, which is more than 6 times longer than that of device E (*T*_90_ = 31 min) under a nitrogen atmosphere. Besides, after examination of the microscopic pictures of the OLED (the inset of Fig. 6a), better resistance to corrosion from water vapor and oxygen, and less dark stains occurred at light-emitting areas of the unencapsulated PhOLEDs.

The reason for the long operating lifetime can be attributed to the minimized interactions between the triplets on the dopant and the polarons on the host molecules. To demonstrate the decrease of the triplet population, the transient decay characteristics of DPDDC doped in a typical CBP host were further investigated. Fig. S12 of the ESI† shows the PL transient decay curves of the Ir-doped CBP films. Table S3 (in the ESI†) summarizes the fitted lifetimes from the Ir-complex films. The main emission of the doped film will harvest from the triplet state of the guest *via* the energy transfer from the host to guest. The decay time of the (mdppy)_2_Iracac emission in the (mdppy)_2_Iracac:CBP film (1.78 μs) is about 0.25 μs higher than that of (mdppy)_2_Iracac:DPDDC film (1.53 μs). Thus, the (mdppy)_2_Iracac:DPDDC system exhibits low triplet density due to the presence of the process of intersystem crossing and reverse intersystem crossing on photoexcited DPDDC.

Previous studies have suggested that the intrinsic degradation of PhOLEDs results from the energetically driven formation of traps that can quench excitons and also act as non-radiative charge recombination centers.^46–48^ These traps are formed due to bimolecular triplet–polaron annihilation where energy is transferred from triplets to polarons. Here, the small Δ*E*_ST_ of DPDDC endowed efficient reverse ISC from triplet to singlet, reducing the triplet density on the host and thus the diminished TPA events. In addition, charges are effectively confined in the emitting layer due to the large energy barrier for hole and electron leakage, which suppresses the degradation of hole and electron transport materials. Also, the high glass transition temperature also has a positive effect on the lifetime because crystallization of the emitting layer can be avoided.

## Conclusions

In conclusion, we have demonstrated an effective strategy of constructing a host material for extremely stable PhOLEDs with low efficiency roll-off. DPDDC, which has an indenocarbazole donor and a 2,4-diphenyl-1,3,5-triazine acceptor linked by an unconjugated benzene ring, has been designed and synthesized. The compound shows excellent hole and electron transport properties, superior thermal stability, high glass-transition temperature, and a small Δ*E*_ST_ for efficient reverse ISC from the triplet to singlet, reducing the triplet density of the host for PhOLEDs. Green PhOLEDs based on the DPDDC host and 5 wt% (tpy)_2_Iracac, (ppy)_2_Iracac, and (mdppy)_2_Iracac emitters exhibited very high external quantum efficiency of 18.1%, 22.6% and 23.6% and extremely low current efficiency roll-off of 2.3%, 5.2% and 5.5% at 5000 cd m^–2^, resulting from the efficient reverse ISC from the triplet to singlet, and thus the subdued TTA and TPA on the host. In addition, our device also presented a high power efficiency of 92.3 lm W^–1^ (84.3 cd A^–1^) and a twentyfold operating lifetime improvement (time to 90% of the 5000 cd m^–2^ initial luminance) compared to a CBP-based device. These make DPDDC a promising host for high performance OLED displays and lighting applications.

## Experimental section

### General information

All chemicals and reagents were used as received from commercial sources without further purification unless stated otherwise. The auxiliary materials for OLED fabrication such as 4,4′,4′′-tris-*N*-naphthyl-*N*-phenylamino-triphenylamine (2T-NATA), *N*,*N*′-bis(1-naphthyl)-*N*,*N*′-diphenyl-[1,1′-biphenyl]-4,4′-diamine (NPB), 4,40,400-tris(*N*-carbazolyl)-triphenyl-amine (TCTA), 1,3,5-tris(*N*-phenylbenzimidazol-2-yl) benzene (TPBi) and lithium quinolinolate (Liq) were purchased from Yurui (Shanghai) Chemical Co. Ltd.

### Synthesis

#### 9,9-Dimethyl-2-(2-nitrophenyl)-9*H*-fluorene (**1**)

2-(9,9-Dimethyl-9*H*-fluoren-2-yl)-4,4,5,5-tetramethyl-1,3,2-dioxaborolane (8.7 g, 27.22 mmol), 1-bromo-2-nitrobenzene (5 g, 24.75 mmol), tetrakis(triphenylphosphine)palladium(0) (0.5 g, 0.43 mmol) and potassium carbonate (10.32 g, 74.26 mmol) with tetrahydrofuran (150 mL) and water (50 mL) solvents were refluxed overnight in a 250 mL round bottom flask. The solution was extracted with dichloromethane, and dried over anhydrous MgSO_4_. After removal of the solvent under reduced pressure, the final residue was purified using column chromatography (eluent, dichloromethane/hexane, 1 : 3) to get the pale yellow solid 9,9-dimethyl-2-(2-nitrophenyl)-9*H*-fluorene (6.2 g, yield 80%).^1^H NMR (CDCl_3_, 500 MHz): *δ* ppm 7.86 (dd, *J*_1_ = 1.0 Hz, *J*_2_ = 8.0 Hz, 1H), 7.76 (m, 2H), 7.63 (dd, *J*_1_ = 1.0 Hz, *J*_2_ = 7.5 Hz, 1H), 7.53 (dd, *J*_1_ = 1.0 Hz, *J*_2_ = 7.5 Hz, 1H), 7.47 (m, 2H), 7.39 (s, 1H), 7.35 (m, 2H), 7.31 (dd, *J*_1_ = 1.0 Hz, *J*_2_ = 7.5 Hz, 1H).

#### 12,12-Dimethyl-11,12-dihydroindeno[2,1-*a*]carbazole (**2**)

A mixture of 9,9-dimethyl-2-(2-nitrophenyl)-9*H*-fluorene (5 g, 15.85 mmol) and triphenylphosphine (10.4 g, 1149.44 mmol) was dried in vacuum and filled with nitrogen gas in a 250 mL round bottom flask. 1,2-Dichlorobenzene (150 mL) was added to dissolve the mixture and refluxed for 12 hours in a nitrogen atmosphere. After solvent removal with distillation, the residue was divided using column chromatography (eluent, CH_2_Cl_2_/hexane, 1 : 7) and then recrystallized with CH_2_Cl_2_/hexane to obtain 12,12-dimethyl-11,12-dihydroindeno[2,1-*a*]carbazole 1.7 g (yield 38%). ^1^H NMR (CDCl_3_, 500 MHz) *δ* ppm: 8.09 (dd, *J*_1_ = 8.5 Hz, *J*_2_ = 8.0 Hz, 3H), 7.81 (d, *J* = 7.5 Hz, 1H), 7.67 (d, *J* = 8.0 Hz, 1H), 7.51 (m, 2H), 7.43 (m, 1H), 7.39 (m, 1H), 7.33 (m, 1H), 7.27 (m, 1H), 1.71 (s, 6H).

#### 11-(3-(4,6-Diphenyl-1,3,5-triazin-2-yl)phenyl)-12,12-dimethyl-11,12-dihydroindeno[2,1-*a*]carbazole (**3**)

A mixture of 12,12-dimethyl-11,12-dihydroindeno[2,1-*a*]carbazole (1 g, 3.53 mmol) and 2-(3-bromophenyl)-4,6-diphenyl-1,3,5-triazine (1.5 g, 3.88 mmol), K_3_PO_4_ (3.3 g, 10.59 mmol), CuI (0.7 g, 3.53 mmol), *trans*-1,2-diaminocyclohexane (0.4 g, 3.53 mmol) in 1,4-dioxane (60 mL) was refluxed for 48 h under a nitrogen atmosphere. After cooling to room temperature, the mixture was extracted using ethyl acetate and distilled water. The organic layer was dried over anhydrous magnesium sulfate and evaporated in vacuum to give the crude product, which was purified using column chromatography on silica gel with dichloromethane/petroleum ether gradient mixture as the eluent, providing a yellow powder 1 g. Yield: 45%. ^1^H NMR (500 MHz, CDCl_3_) *δ* ppm: 9.06 (s, 1H), 8.92–8.90 (m, 1H), 8.79–8.77 (m, 4H), 8.51 (s, 1H), 8.27 (d, *J* = 7.5 Hz, 1H), 7.91–7.86 (m, 3H), 7.62–7.51 (m, 8H), 7.47–7.40 (m, 3H), 7.37–7.30 (m, 2H), 1.56 (s, 6H). HRMS calculated for C_42_H_30_N_4_ 590.2470, found: 591.2459 [M + H]^+^.

### Measurements

^1^H NMR and ^13^C NMR spectra were recorded on a Bruker AV-500 spectrometer at room temperature. High resolution mass spectra (HRMS) were determined on a Thermo Fisher Scientific LTQ FT Uitra mass spectrometer. UV-vis absorption spectra were recorded on a UV-2501PC instrument. Photoluminescence spectra were taken using a FLSP920 fluorescence spectrophotometer, both in solution and in the solid state. Cyclic voltammetry and differential pulse voltammetry were carried out using a CH Instrument 660E electrochemical analyzer and with a Ag/AgCl electrode as the reference electrode, with tetra(*n*-butyl)ammonium hexa-fluorophosphate (TBAPF_6_) in DMF as the supporting electrolytes. The glass transition temperatures (*T*_g_) of the compounds were determined under a nitrogen atmosphere using differential scanning calorimetry on a TA Q500 HiRes thermal analyzer with a scanning rate of 10 °C min^–1^ with nitrogen flushing. The decomposition temperature corresponding to 5% weight loss was conducted on a TA Q500 HiRes TGA thermal analyzer.

### Device fabrication

The devices were fabricated using conventional vacuum deposition of the organic layer and cathode onto an indium-tin-oxide (ITO) coated glass substrate under a base pressure lower than 5.0 × 10^–5^ mbar. Prepared glass substrates were cleaned using detergent, de-ionized water, acetone, and isopropanol. Immediately prior to loading into a custom-made high vacuum thermal evaporation chamber, the substrates were treated to a UV-ozone environment for 15 min. Then, organic layers and a metal cathode layer were evaporated successively by using shadow mask. The Ir-complexes in the Host-006 films were deposited on the glass substrates through thermal evaporation under a 5.0 × 10^–5^ mbar pressure to determine the PL spectrum and material characteristics. The entire organic layers and the Al cathode were deposited without exposure to the atmosphere. The deposition rates for the organic materials, and Al were typically 1.0, and 5.0 Å s^–1^, respectively.

### Device measurement

The current density–voltage–luminescence (*J*–*V*–*L*) characteristics were measured using a Keithley 2400 source meter and a PR-650 Spectra Colorimeter. The luminance and spectra of each device were measured in the direction perpendicular to the substrate. The lifetime of the bare devices was performed under a dry-nitrogen atmosphere (<1 ppm H_2_O and O_2_) and in ambient air (∼20% humidity).
