# Supplementary material for: Stable green phosphorescence organic light-emitting diodes with low efficiency roll-off using a novel bipolar thermally activated delayed fluorescence material as host
Source: Chem Sci. 2016 Oct 4;8(2):1259–68. doi: 10.1039/c6sc03008d (PMC5369523; doi:10.1039/c6sc03008d)
Supplement: Supplementary file 1 [file SC-008-C6SC03008D-s001.pdf]

Electronic Supplementary Information

Stable Green Phosphorescence Organic Light-Emitting Diodes with Low Efficiency Roll-off using a Novel Bipolar Thermally Activated Delayed Fluorescence Material as Host

Kunping Guo,‡[ab] Hedan Wang,‡[bc] Zixing Wang,*[b] Changfeng Si,[b] Cuiyun Peng,[a] Guo Chen,[b] Jianhua Zhang,[b] Gaofeng Wang[d] and Bin Wei*[ab]

[a] *School of Mechatronic Engineering and Automation, Shanghai University, 149 Yanchang Road, Shanghai, 200072, P. R. China.*

[b] *Key Laboratory of Advanced Display and System Applications, Ministry of Education, Shanghai University, 149 Yanchang Road, Shanghai, 200072, P. R. China.*

**E-mail:* zxwang78@shu.edu.cn;bwei@shu.edu.cn.

[c] *Department of Chemistry, Shanghai University, 99 Shangda Road, Shanghai, 200444, P. R. China.*

[d] *Ningbo Intime Technology Co. Ltd, No. 23, Ruhu West Road, Simen Town, Yuyao City, Zhejiang Province, 345403, P. R. China*

‡ These authors contributed equally to this work.

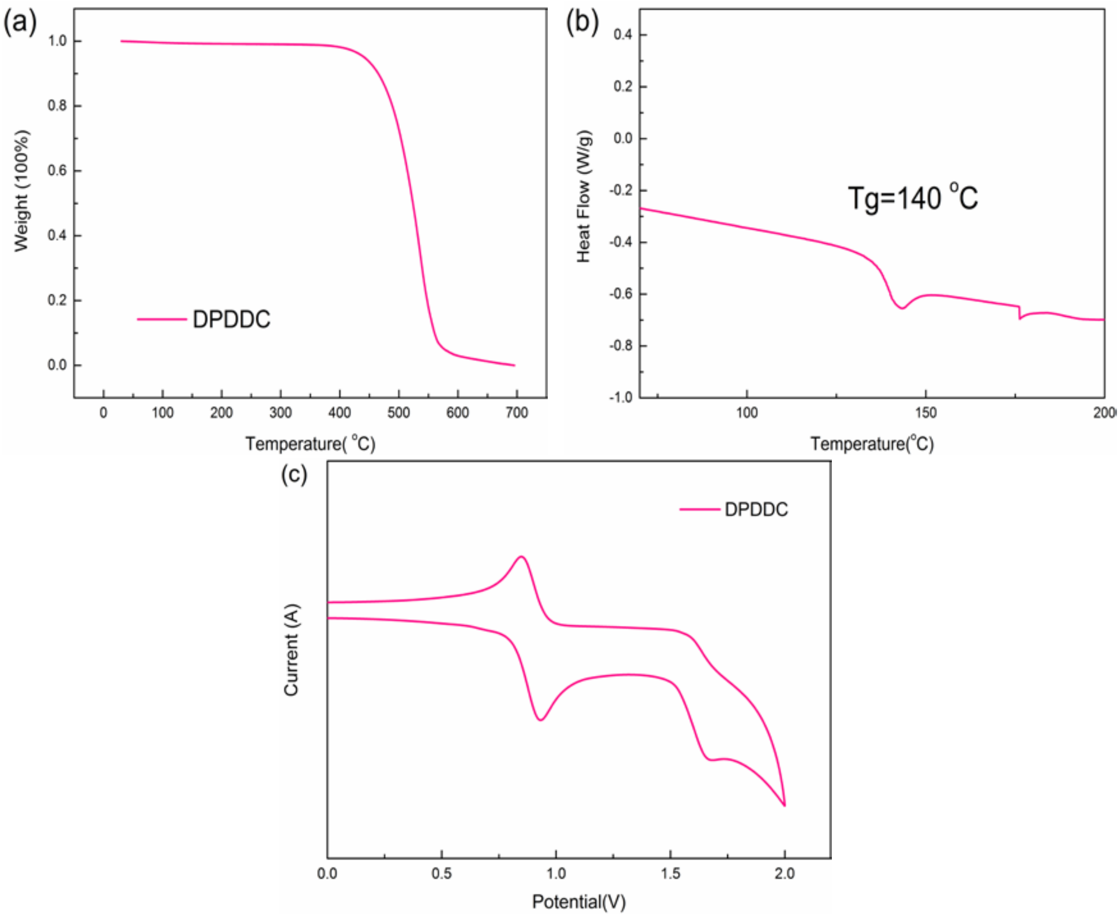

Fig. S1 TGA (a), DSC (b) and Cyclic voltammograms (c) of DPDDC.

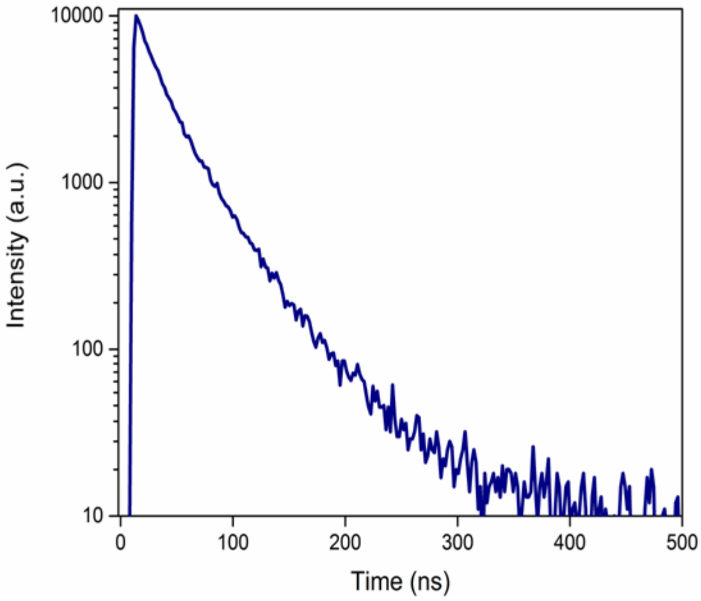

Fig. S2 Photoluminescence decay curve of 10 wt% DPDDC:mCP film in the time range of 500 ns.

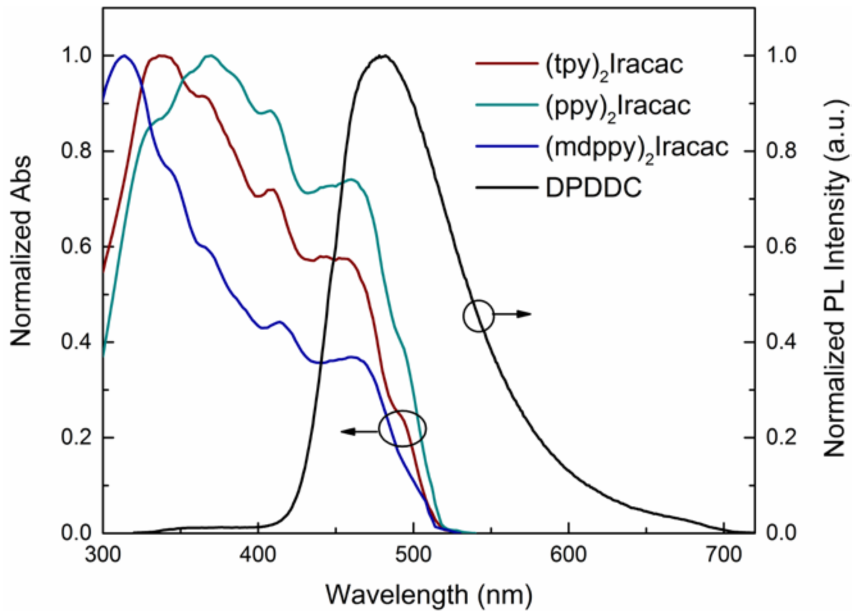

Fig. S3 The overlap between absorption spectrum of acceptor and PL spectrum of donor, the acceptor is $(tpy)_2$Iracac, $(ppy)_2$Iracac or $(mdppy)_2$Iracac and the donor is DPDDC, the overlap indicates efficient Förster energy transfer.

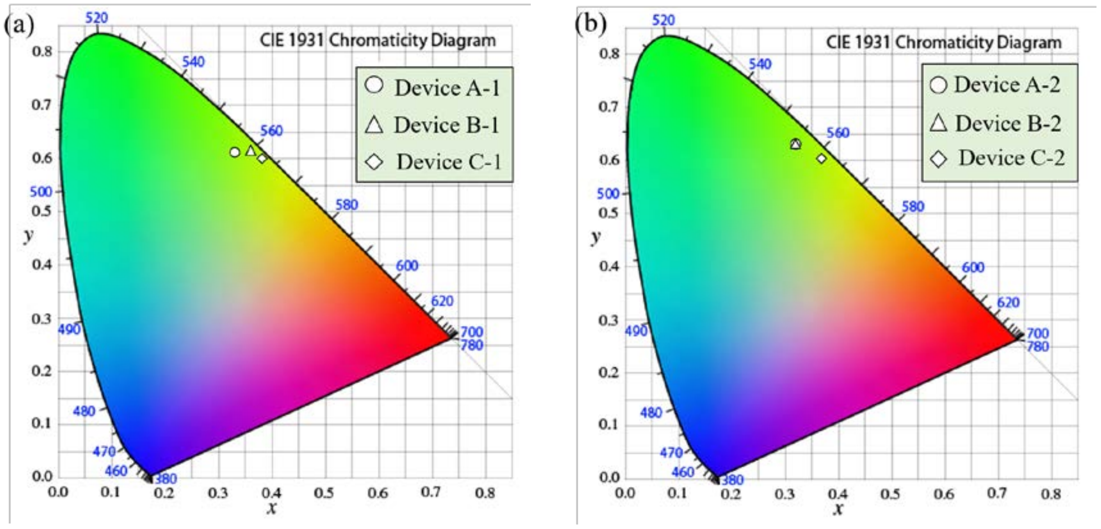

Fig. S4 The CIE 1931 chromaticity coordinates of Devices A-1, B-1, C-1 (a) and Devices A-2, B-2, C-2 (b).

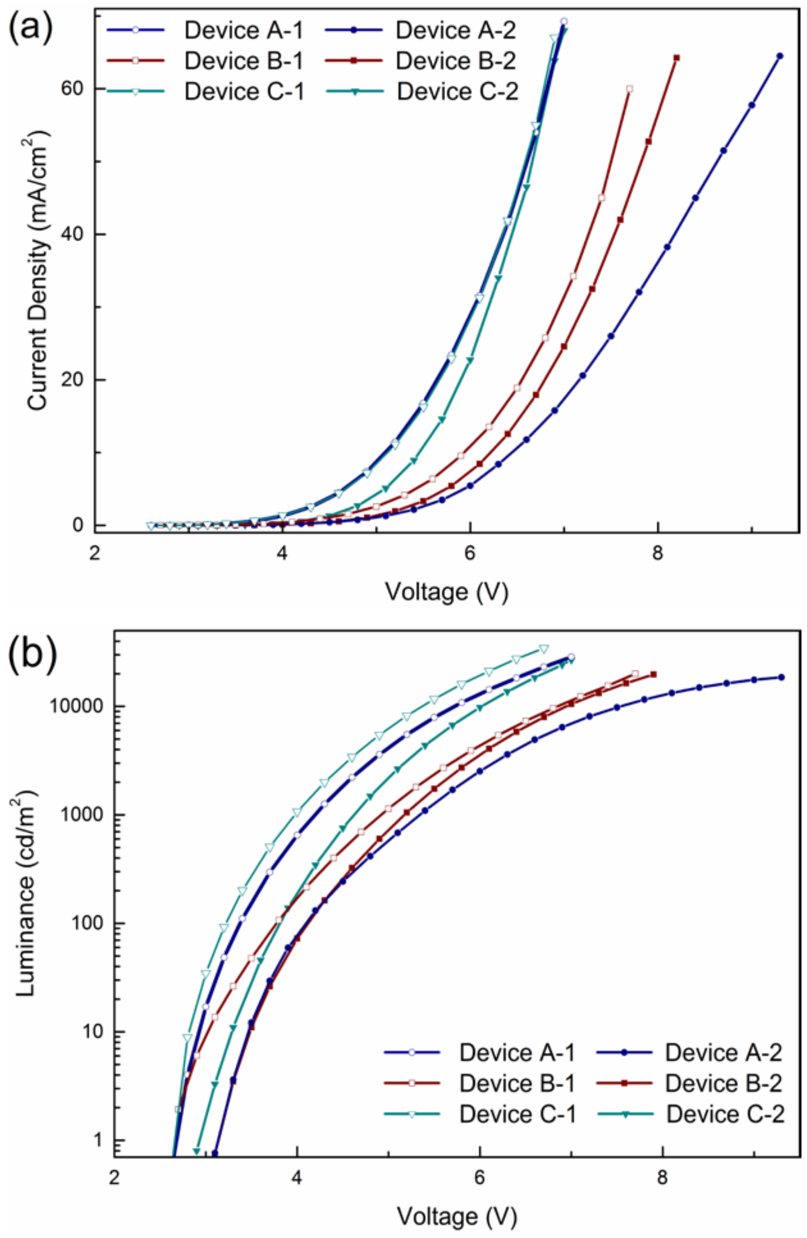

Fig. S5 Current density-voltage characteristics (a), luminance-voltage characteristics (b) of the Devices.

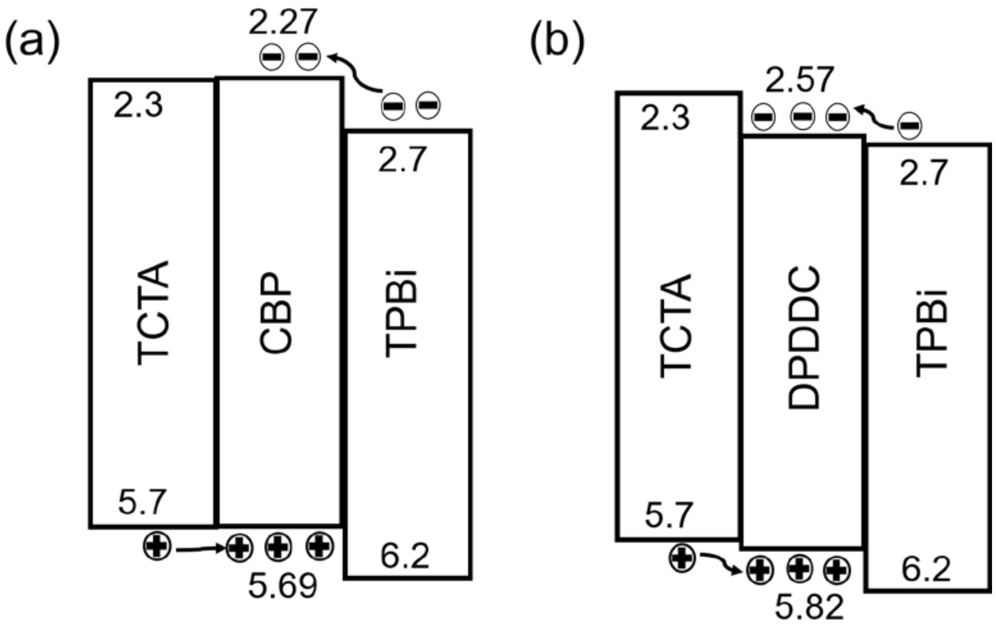

Fig. S6 Energy levels of EML and transport layers adjacent to EML with CBP (a) and DPDDC (b) being host of EML.

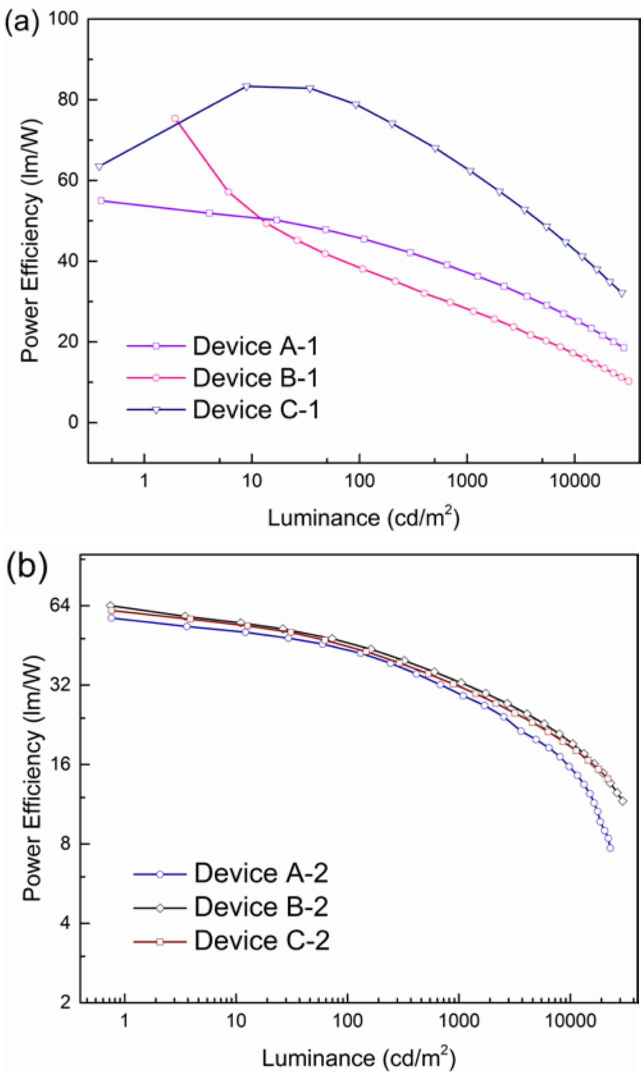

Fig. S7 Power efficiency plotted luminance for Devices A-1, B-1, C-1 (a) and Devices A-2, B-2, C-2 (b).

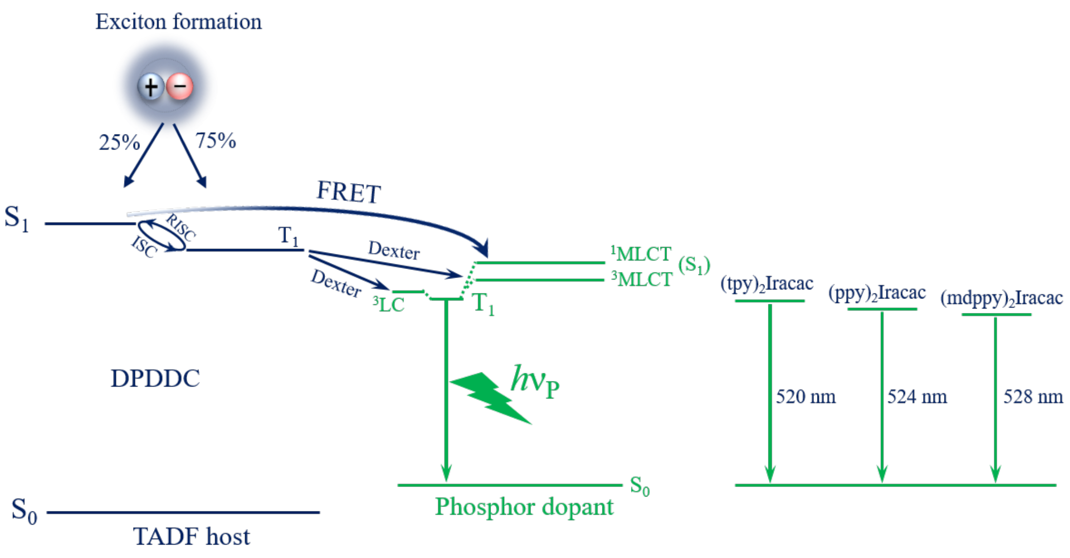

Fig. S8 Schematic diagram of emission process in the PhOLEDs based on the DPDDC host.

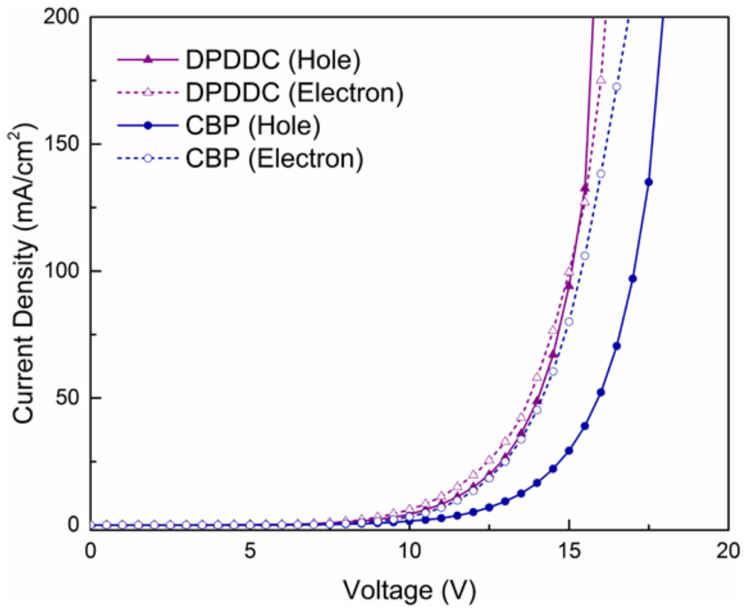

Fig. S9 *J*-*V* characteristics of hole-only and electron-only devices.

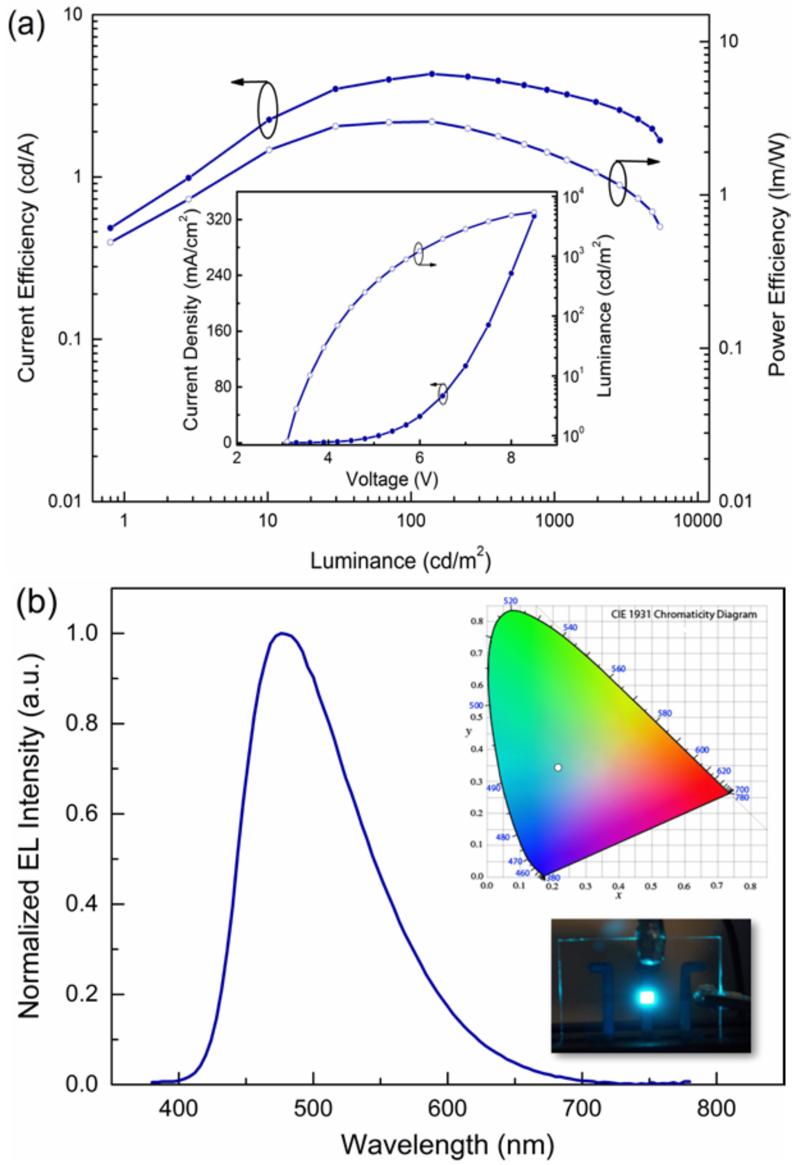

Fig. S10 (a) Current efficiency and power efficiency plotted against luminance for Device D. Inset is current density-voltage and luminance-voltage characteristics curves of Device D; (b) EL spectra of Device D at 1000 cd m^{-2}. The top right inset shows CIE chromaticity coordinates of Device D and the lower right inset shows the photo of the test Device D.

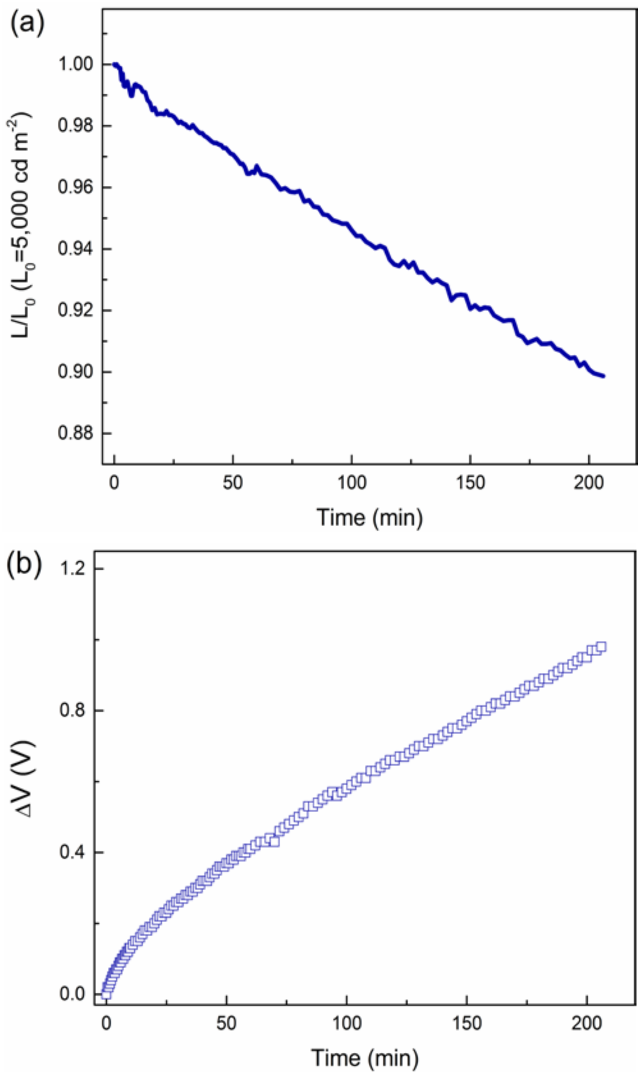

Fig. S11 Time evolution of the normalized luminance, L, of Devices C-3 in ambient air (~20% humidity) atmosphere (a) and change in operating voltage ΔV (offset to zero) at the initial luminance of L_0 = 5,000 cd m^{-2} (b).

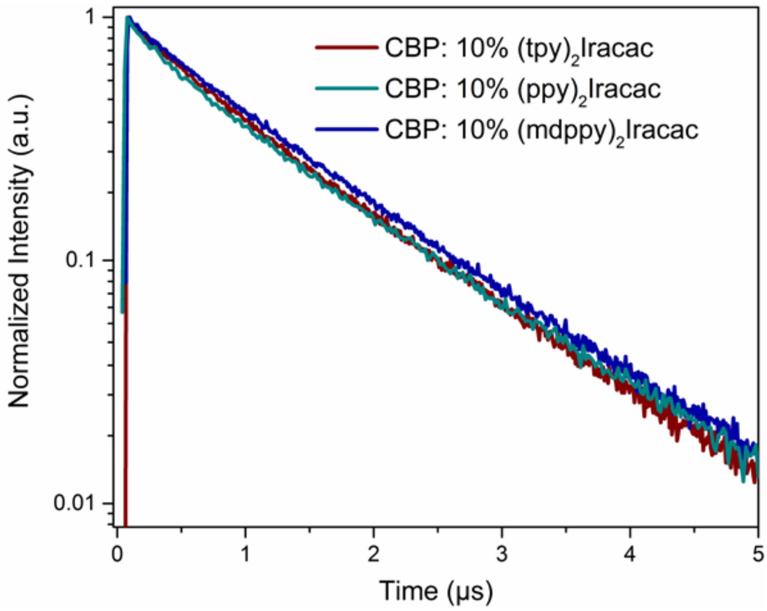

Fig. S12 PL transient decay curves of Ir-doped CBP films.

Table S1 Physical Properties of DPDDC

host	$\lambda_{abs\ Sol}$[a] [nm]	$\lambda_{PL\ Sol}$[a] /film[b] [nm]	λ_{Ph}[c] [nm]	E_{ox}[d] [V]	E_{red}[d] [V]	HOMO/LUMO [eV]	E_g[g] [eV]	E_T[h] (eV)	T_g/T_d[i] [℃]
DPDDC	308,345,360	515/475	445	1.02	-2.73	-5.82/-2.57[e]<br>-5.30/-2.06[f]	3.25	2.79	140/441

[a] $\lambda_{abs\ Sol}$, $\lambda_{PL\ Sol}$ measured in 2-Methyl-THF solutions at room temperature. [b] λ_{PL} film, measured in thin solid film. [c] Measured in 2-Methyl-THF solutions at 77K. [d] E_{ox} = oxidation potential and E_{red} = reduction potential was determined by DPV. [e] The HOMO and LUMO values were determined from the oxidation/reduction potential from CV curves. [f] Values from DFT calculation. [g] The value of E_g was calculated from the absorption onset of high concentration. [h] The value of E_T was estimated from the peak values of phosphorescence spectra measured in 2-Methyl-THF solutions at 77K. [i] T_g: glass transition temperatures, T_d: decomposition temperatures of 5% weight loss, Obtained from DSC and TGA measurements.

Table S2 The excitation energies of Ir-complexes, DPDDC and CBP.

Excitation energy	CBP	DPDDC	$(tpy)_2Iracac$	$(ppy)_2Iracac$	$(mdppy)_2Iracac$
Singlet (eV)	3.63	2.98	2.71 (^{1}MLCT)	2.69 (^{1}MLCT)	2.67 (^{1}MLCT)
Triplet (eV)	2.81	2.79	2.53	2.55	2.48

Table S3 The fitted lifetimes from Ir-complex films.

Lifetime (μs)	10 wt% Ir-complex		
	$(tpy)_2$Iracac	$(ppy)_2$Iracac	$(mdppy)_2$Iracac
CBP	1.62	1.67	1.78
DPDDC	1.52	1.49	1.53

PL characteristics and rate constants of TADF molecules.

The rate constants were calculated using the equations described in Ref. 22 with the measured PL efficiencies and decay times:

$$\tau_p = 1/\kappa_p, \quad \tau_d = 1/\kappa_d \tag{1}$$

$$\kappa_r^S = \phi_{prompt}\kappa_p \tag{2}$$

$$\kappa_{nr}^T = \kappa_d - \phi_{prompt}\kappa_{RISC} \tag{3}$$

$$\kappa_{ISC} = (1-\phi_{prompt})\kappa_p \tag{4}$$

$$\kappa_{RISC} = \frac{\kappa_p \kappa_d}{\kappa_{ISC}} \frac{\phi_{delayed}}{\phi_{prompt}} \tag{5}$$

where τ_p is the transient decay time of the prompt component, τ_d is the transient decay time of the delayed component, κ_p is the transient decay rate of the prompt component, κ_d is the transient decay rate of the delayed component, κ_r^S is the radiative decay rate from the S_1 state to the ground state, κ_{nr}^T is the radiative decay rate from the T_1 state to the ground state, κ_{ISC} is the rate constant of ISC, κ_{RISC} is the rate constant of RISC, and $\phi_{delayed}$ and $\phi_{delayed}$ are the prompt and delayed components of the PL quantum efficiency, respectively.
